# The zinc finger domains of PARP‐1 are selectively and potently inhibited by the Au(I)‐based drugs sodium aurothiomalate and aurothioglucose

**DOI:** 10.1002/1873-3468.70224

**Published:** 2025-11-13

**Authors:** Uliana Bashtanova, Melinda Jane Duer

**Affiliations:** ^1^ Yusuf Hamied Department of Chemistry University of Cambridge UK

**Keywords:** auranofin, aurothioglucose, aurothiomalate, Poly(ADP‐ribose) polymerase, synthetic lethality, zinc finger

## Abstract

Poly(ADP‐ribose) polymerase‐1 (PARP‐1) is a multidomain enzyme essential for the DNA damage response; its inhibition can lead to cancer cell death. Recruitment of PARP‐1 to sites of genomic damage is mediated by its zinc finger domains. In this study, we investigated the inhibition of PARP‐1's DNA‐dependent activation by three Au(I)‐based drugs, presumable zinc‐ejectors. We found that aurothioglucose and sodium aurothiomalate selectively inhibited PARP‐1's DNA‐dependent activity, with IC_50_ values in the nanomolar range, while preserving its DNA‐independent activity. Furthermore, in a BRCA‐mutated cell line, both compounds effectively suppressed DNA replication, with half‐maximal effective concentrations (EC_50_) also in the nanomolar range. These findings highlight the potential of selective, zinc finger–targeting PARP‐1 inhibitors as promising candidates for anticancer drug testing.

**Impact statement**

The need to preserve basal poly(ADP‐ribose) polymerase‐1 (PARP‐1) activity in healthy cells, along with the search for alternative anticancer therapies in cases of drug resistance, led to the discovery of selective and potent zinc finger–targeting inhibitors of PARP‐1. This finding opens new opportunities for anticancer drug development.

## Graphical abstract

PARP‐1 is a key enzyme in the DNA damage response, and its inhibition induces cancer cell death via synthetic lethality. Au(I)‐based drugs, such as aurothioglucose and sodium aurothiomalate, block PARP‐1's DNA‐dependent activity by targeting its zinc finger domains. Importantly, the catalytic site remains unaffected, preserving DNA‐independent functions in healthy cells.

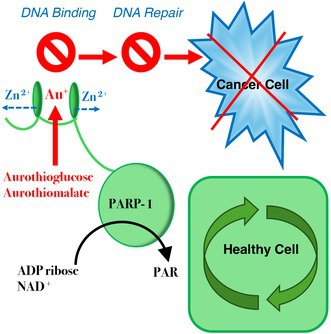

## Abbreviations

**ADPR**, ADP‐ribose

**BrdU**, 5‐Bromo‐2'‐deoxyuridine

**NAM**, nicotinamide

**PAR**, Poly(ADP‐ribose)

**PARP**, Poly(ADP‐ribose) polymerase

Poly (ADP‐ribose) polymerases (PARPs) are multidomain enzymes essential to multiple cell processes. They cleave NAD^+^ to nicotinamide (NAM) and ADP‐ribose (ADPR) and add an ADPR unit either to an acceptor protein, resulting in mono ADP ribosylation, or to an existing PAR chain resulting in poly ADP ribosylation (PARylation) of the acceptor protein. There are 17 PARP enzymes in humans [1] of which only PARP‐1, PARP‐2, and tankyrase 1 and 2 are known to catalyze PARylation [2]. In the cell, most of the stimulated and basal PARP activity is exerted by PARP‐1 (85–90%) and PARP‐2 (10–15%) [3].

The most‐documented role of PARP‐1 and PARP‐2 is in DNA damage repair (DDR) [4, 5]. Recent findings suggest that PARP‐2 is unlikely to be recruited to sites of DNA damage without initial PARylation by PARP‐1 [6]. Thus, the action of PARP‐1 in DNA repair becomes decisive for cell survival when there is a pre‐existing mutation in the double–strand repair mechanism, such as mutations in BRCA1/2 and PALB2. In such cases, inhibition of PARP‐1 ensures elimination of cells carrying the mutations [7]. This has made PARP‐1 a target of interest in cancer therapeutics, and the discovery of small molecule inhibitors of PARP‐1 has recently resulted in the approval of ‘parib’ drugs such as olaparib, rucaparib, veliparib, and talazoparib for different stages of breast and ovarian cancers.

The list of known PARP cellular activities reaches far beyond DNA repair; however, it includes protein degradation, protein acetylation, stress signaling, apoptosis, redox defense, immune response, viral infection (reviewed in [8]), and biomineralization [9, 10]. Thus, despite the parib drugs having a very precise mechanism of action, they still demonstrate a toxicity profile [11], probably due to less prominent, but essential roles of PAR in cellular and tissue physiology.

The recruitment of PARPs to sites of genomic damage occurs via zinc fingers and/or the WGR domain, with DNA‐binding zinc finger domains being a unique feature of PARP‐1. To activate PARP‐1, two zinc finger (F) domains—F1 and F2—act cooperatively [12]. The third zinc‐binding domain in PARP‐1, F3, contains a unique type of zinc‐ribbon fold structure, in which several amino acids are crucial for the DNA‐dependent PARP‐1 hyperactivation, but dispensable for the basal PAR synthesis [5]. Thus, targeting the PARP‐1 zinc fingers seems a direct path to inhibit DNA repair while preserving the basal catalytic activity of PARP‐1. We explore in this paper the PARP‐1 inhibition potential of three Au (I) drugs used to treat rheumatoid arthritis—aurothioglucose, sodium aurothiomalate and auranofin. Au (I) and (III) have been reported to interact with cysteine residues of various zinc fingers leading to Zn^2+^ displacement and formation of the so‐called gold fingers.

## Material and methods

### PARP enzyme activity assay

PARP‐1 and PARP‐2 activity and inhibition were measured using the PARP1‐Enzyme‐Activity‐Assay as a direct fluorescence‐based concentration measurement of reaction product formation. The assay reagents are sold as a commercial kit (17‐10149; Merck Millipore, Burlington, MA, USA). To measure PARP inhibition, the NAD+ substrate concentration was set at or below *K*_m_ (the Michaelis constant) to enable identification of all types of inhibitors competitive, uncompetitive, and noncompetitive (allosteric) [13], the latter represents a mode of action of zinc‐finger inhibitors. PARP activity and inhibition were measured for human full‐length recombinant active PARP‐1 (CS207770; Merck Millipore, Burlington, MA, USA) and PARP‐2 (ab198766; Abcam, Cambridge, UK).

Four compounds (auranofin; (Bio‐Techne, Minneapolis, MN, USA), sodium aurothiomalate, aurothioglucose (1–100 000 nm), and PJ34 (5–50 000 nm) (Sigma‐Aldrich, St. Louis, MO, USA)) at six concentrations were added to the reaction buffer, formulated as a 1 : 1 mixture of the kit buffer and the lab‐made buffer (50 mm Tris–HCl, 100 mm NaCl, 5 mm MgCl_2_, 0.05% Tween‐20, pH 8.0, all reagents from Sigma), and incubated either with PARP‐1 (2.5 ng·μL^−1^ final) or PARP‐2 (2.2 ng·μL^−1^ final) at room temperature for 30 min. Activated DNA (2 ng·μL^−1^ final; Merck Millipore kit), β‐NAD (60 μm final; Merck Millipore kit), and nicotinamidase (200 ng·μL^−1^ final, Merck Millipore kit) were added and incubated at 37 °C for 45 min. Final reaction volume was 25 μL.

Controls were as follows:

1. control of 0% inhibition contained reaction sample without inhibitor;
2. control of 100% inhibition of PARP activity contained reaction sample without β‐NAD; and
3. control of 100% inhibition of DNA‐dependent PARP activity contained reaction sample without DNA but with β‐NAD.

After the plates were cooled down to room temperature, 25 μL of Merck Millipore kit proprietary reagent was added to the reaction mixture and incubated with mild shaking for 45 min. Fluorescence measurement was carried out at an excitation wavelength of 410 nm and an emission of 460 nm in a Fluostar Omega microplate reader (BMG Labtech, Ortenberg, Germany).

### 5‐Bromo‐2′‐deoxyuridine (BrdU) incorporation assay

BrdU was incorporated into the DNA of replicating UWB1.289 cells by substituting thymidine, and BrdU‐containing DNA was subsequently detected with BrdU‐specific antibodies. Assay execution was subcontracted to Cellomatics Biosciences, UK. At Day 0, cells were split and seeded into a 96 well‐plate at around 500 cells/well. At Day 1, cell adherence was confirmed, and media were changed to include six compounds (rucaparib, olaparib, auranofin (0.01–10 000 nm), talazoparib (0.005–5000 nm), sodium aurothiomalate (0.05–50 000 nm), and aurothioglucose (1.2–120 000 nm)) at seven concentrations, in triplicates. The media also contained BrdU 1X (Roche, Basel, Switzerland). Then, cells were incubated at 37 °C, 5% CO_2_, for 6 days, allowing cumulative cell proliferation to be measured over this period. After 6 days of incubation, the media were discarded, and cells were fixed for 30 min, at room temperature, with FixDenat Solution, (Roche) and substituted with Anti‐BrdU‐POD working solution (Roche) for 2 h at room temperature. Plates were washed three times with washing buffer (Roche), and substrate solution (Roche) was added. The reaction was stopped by adding H_2_SO_4_ and absorbance was immediately read at 450 nm. The absorbance average and the standard error were calculated using the technical triplicate for each condition. The percentage of DNA replication was calculated as: % = 100% × (Absorbance treated)/(Absorbance untreated), reflecting relative cell proliferation over the 6‐day period; the untreated cells were assumed to demonstrate 100% DNA replication activity. Blanks were represented by wells with no cells and cells without BrdU; both showed no signal.

### CellTiter Glo luminescence cell viability assay

The CellTiter‐Glo^®^ Luminescent Cell Viability Assay (Promega, Madison, WI, USA) is a homogeneous method of determining the number of viable cells in culture based on quantitation of the ATP present, an indicator of metabolically active cells. At Day 0, cells were split. HCC1937 cells were seeded into a 384‐well plate at around 1000 cells/well. Vascular smooth muscle cells were seeded into a 96‐well plate at 2000 cells/well. At Day 1, cell adherence was confirmed. Then, the following compounds were added to HCC1937 cells (rucaparib, olaparib, and talazoparib, aurothioglucose (1.52–30 000 nm) auranofin, sodium aurothiomalate, doxorubicin, and paclitaxel (10–10 000 nm)) at 7–10 concentrations, in triplicates. To vascular smooth muscle cells compounds (talazoparib, aurothioglucose, sodium aurothiomalate (1.3–130 000 nm), auranofin (1.4–140 000 nm), and paclitaxel (1.2–120 000 nm)) were added at six concentrations, in quadruplicates. Plates were incubated at 37 °C, 5% CO_2_ for 3–6 days. CellTiter‐Glo reagent (Promega) was thawed to room temperature and added to each well (at a 1 : 1 ratio to the culture medium) after a plate was equilibrated for 15 min at room temperature. After 10‐min incubation with reagents, plates were read by PHERAstar FS Plate Reader (BMG Labtech, Ortenberg, Germany). The percent viability was calculated as: % = 100−(100 × (Luminescence from cells treated with vehicle ‐Luminescence from cells treated with drug)/(Luminescence untreated – Luminescence blank)).

### Statistics

To obtain IC_50_ and EC_50_ values, the data were fitted with sigmoidal curves in Origin‐ using either dose–response or logistic models, depending on the presence or absence of pronounced plateaus in the data. The central slope value was a fitted parameter that corresponded to the IC_50_ or EC_50_, and their standard errors of the mean (s.e.m.) were also calculated by Origin™ during the fitting process.

### Cell cultures

UWB 1.289 (ATCC CRL‐2945, RRID:CVCL_B079) and HCC1937 (ATCC CRL‐2336, RRID:CVCL_0290) cell lines were obtained from ATCC and cultivated according to the handling instructions. These cell lines have been authenticated using short tandem repeat (STR) profiling, and confirmed free from contamination with other cell lines and mycoplasma in the past three years. The bovine aorta vascular smooth muscle cells (BVSMC) were obtained from Prof. Catherine Shanahan, King's College London. Passage # 7 was used for the experiments. Cells were grown in Dulbecco's Modified Eagle Medium (DMEM; Gibco, Thermo Fisher Scientific, Waltham, MA, USA) supplemented with 4.5 4.5 g·L^−1^ glucose 10% fetal bovine serum (Pan‐Biotech, Aidenbach, Germany) and 1% L‐Penicillin–Streptomycin‐Glutamine solution (Gibco).

## Results

### Detection of DNA‐dependent and ‐independent activity of PARP‐1

The majority of PARP assays reported in the literature either utilized fluorescently labeled or biotinylated NAD^+^, both of which significantly changed the NAD^+^ binding properties [5, 14], or necessitated zero or very low NAD^+^ concentrations [15, 16, 17], so the kinetic parameters generated were not necessarily representative of those *in vivo*. For our work, we specifically chose an assay capable of measuring PARylation in solution at cellular NAD^+^ concentrations. Intracellular NAD^+^ was known to be unevenly distributed within the cell: in the mitochondria, NAD^+^ concentration is estimated to be 400–1000 μm, while in the nucleus, it was about 100 μm [18, 19]; the latter correlated well with the *K*_m_ value of PARP‐1 previously reported in the literature 86 μm; [5].

**Fig. 1:**
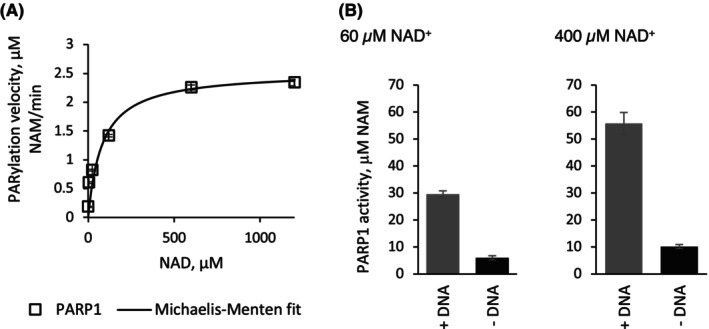
Apparent *K*_m_ of recombinant poly(ADP‐ribose) polymerase‐1 (PARP ‐1) and its activation by single strand break DNA. Poly ADP ribosylation (PARylation) activity of recombinant human full length active PARP‐1 (Merck) enzyme was measured via fluorescence detection of the PARylation by‐product nicotinamide after its conversion into ammonia by the coupled enzyme nicotinamidase (assay kit Merck). Data analysis and fitting were performed in Origin™. Values given as mean ± SE. (A) *K*_m_ was measured at 14 ng·μL^−1^ PARP‐1 in triplicate for each NAD^+^ concentration. For PARP‐1, Vmax was 2.54 ± 0.01 μm NAM/min and apparent *K*_m_ 82 ± 1 μm, *R* ^2^ = 0.99. (B) Activation of PARP ‐1 was measured in the presence or absence of 2 ng·μL^−1^ activated DNA at two NAD^+^ concentrations. Four to eight independent experiments were performed for each condition, with four chemical replicates per experiment. Differences in activation observed for PARP‐1 were statistically significant at a confidence interval of 99% (*t*‐test).

The PARP assay kit designed by Merck (assay kit # 17‐10149; Merck, USA) detects PARylation via the by‐product nicotinamide after its conversion into ammonia by nicotinamidase. This assay enabled us to match the experimental NAD^+^ concentration to that of the nucleus, thus mimicking the chemical environment for the PARP‐1 enzyme localized there. We found that the apparent *K*_m_ value was 82 ± 1 μm for PARP‐1 (Fig. 1A) and matched that reported in the literature 86.5 ± 39.0 μm; [5].

We then tested the level of enzyme activation by DNA at two NAD^+^ concentrations, 60 and 400 μM, to mimic diverse intracellular environments. As expected, PARP‐1 activity was massively activated in the presence of ssb DNA, and the average level of activation was 500% Fig. 1B and [20]. Regardless of the absolute activity, which depended on the NAD^+^ concentration (Fig. 1B), the ratio of DNA‐dependent to DNA‐independent (basal) activity remained constant at 5:1.

### Au (I)‐based drugs are selective and potent PARP‐1 zinc‐finger inhibitors

We hypothesized that Au(I) could replace Zn^2+^ in the zinc fingers of PARP‐1, thereby reducing their affinity for ssb DNA and consequently inhibiting the DNA‐dependent activation of PARP‐1. To test this, we examined the inhibition of PARP‐1 activity by three well‐known and widely used Au(I) drugs—aurothioglucose, auranofin, and sodium aurothiomalate—which are expected to bind zinc fingers.

**Fig. 2:**
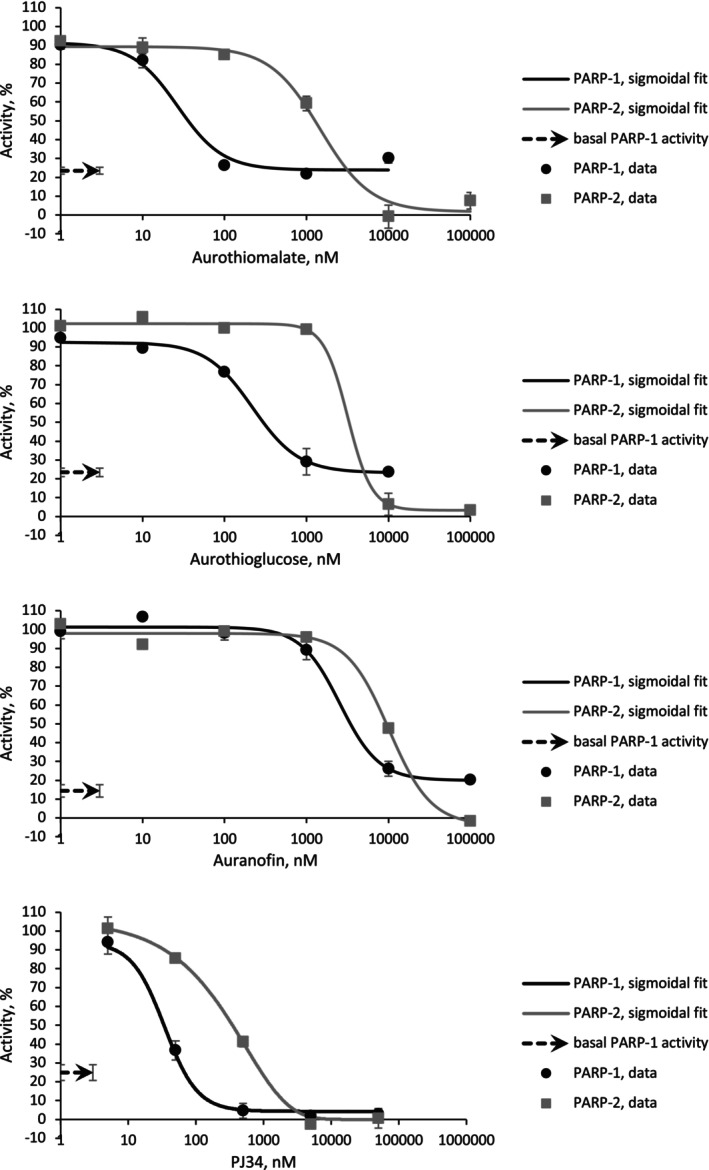
Inhibition of poly(ADP‐ribose) polymerase‐1 and ‐2 (PARP‐1 and PARP‐2) activity by Au (I)‐based drugs and PJ34. PARP‐1 and PARP‐2 activity was measured using a fluorescence‐based assay that detects the PARylation by‐product nicotinamide, following its enzymatic conversion to ammonia by nicotinamidase (assay kit # 17‐10149, Merck, USA). Activity values were converted into percentages according to the controls: a sample without an inhibitor was assigned as 100% activity, and a sample without β‐NAD^+^ as 0% activity. Inhibition of PARP‐1 by PJ34 readily reached 0% activity, but for the Au(I) drugs the inhibition plateaued at around 20%, which was the inhibition level of the control without ssb DNA (basal PARP‐1 activity). Treatments of PARP‐2 with the Au(I) drugs and PJ34 resulted in full inhibition of enzyme activity. Two to four independent experiments were performed in duplicates for each enzyme/compound. Fitting was performed in Origin™, *R* ^2^ ≥ 0.9. Data values are given as mean ± s.e.m.

As shown in Fig. 2, treatment with aurothioglucose and sodium aurothiomalate reduced PARP‐1 activity to the level of the ‘DNA‐absent’ control, plateauing at approximately 20% residual activity rather than reaching complete inhibition (0% activity). This indicates that these two drugs selectively and potently inhibit the DNA‐dependent activity of PARP‐1 in the nanomolar concentration range, while preserving the DNA‐independent (basal) activity of the enzyme (Fig. 2 and Table 1). These findings suggest that the inhibition occurs through disruption of zinc finger–DNA interactions, rather than through direct inhibition of the catalytic site (Fig. 1).

**Table 1:** Aurothioglucose and sodium aurothiomalate are highly potent inhibitors of PARP‐1, rather than PARP‐2, in contrast to PJ34—a potent inhibitor of both PARP‐1 and PARP‐2. Half maximal inhibitory concentration (IC_50_) values of three Au (I)‐based drugs and PJ34 were obtained from fitted curves provided in Fig. 2. For each enzyme/compound, three independent experiments were performed in duplicates. Values are given as mean ± s.e.m.

Enzyme	IC_50_/nm			
	PJ34	Sodium aurothiomalate	Aurothioglucose	Auranofin
PARP‐1	34.4 ± 0.1	24.8 ± 2	206 ± 28	2554 ± 0.2
PARP‐2	383 ± 1	1259 ± 8	2966 ± 11	8986 ± 44

For comparison, we also evaluated the effect of PJ34 [21], a well‐characterized NAD^+^‐mimetic inhibitor of the catalytic site of both PARP‐1 and PARP‐2. As expected, PJ34 in the nanomolar range fully inhibited both enzymes, eliminating DNA‐independent activity as well (Fig. 2).

Auranofin, such as aurothioglucose and sodium aurothiomalate, inhibited PARP‐1 activity down to the level of basal (DNA‐independent) activity, suggesting it also acts as a DNA binding inhibitor. However, auranofin was less potent, with an IC_50_ in the micromolar range (Fig. 2 and Table 1). Overall, all three Au(I) compounds appear to function as zinc finger inhibitors of PARP‐1, with relative potency in the order: sodium aurothiomalate > aurothioglucose > auranofin (Table 1).

To assess specificity, we compared PARP‐1 inhibition curves to those of PARP‐2, which lacks zinc fingers and thus served as a control for zinc finger‐independent inhibition by Au(I)‐based drugs. While PARP‐2 could be fully inhibited by the three compounds, this only occurred at much higher concentrations, suggesting a different mechanism of inhibition, likely unrelated to zinc displacement. The IC_50_ values for PARP‐2 were in the micromolar range or higher, indicating that the Au(I) drugs were neither potent nor specific inhibitors of PARP‐2, as expected (Fig. 2 and Table 1).

### Au (I) drugs effectively inhibit DNA replication in a BRCA‐mutated cell line

**Fig. 3:**
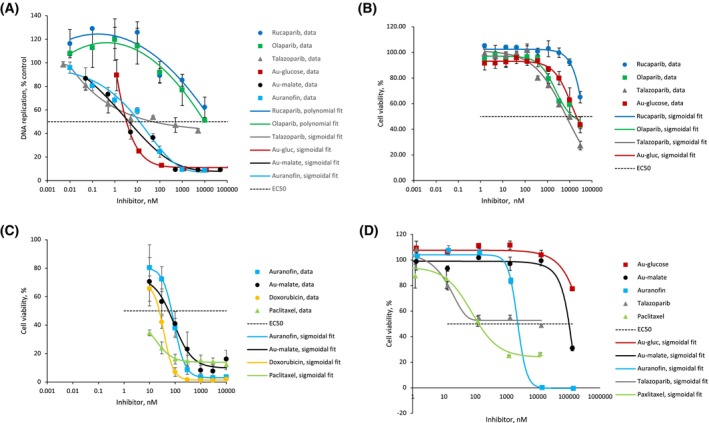
Response of two BRCA‐mutation cell lines with differing drug resistance and primary bovine vascular smooth muscle cells to NAD^+^‐mimetic inhibitors and poly(ADP‐ribose) polymerase‐1 (PARP‐1) zinc finger inhibitors. Dose–response curves were fitted in Origin™, *R* ^2^ ≥ 0.9. Data values are given as mean ± s.e.m. (A) DNA replication in the ‘parib’‐sensitive UWB 1.289 cell line was assayed by bromodeoxyuridine (BrdU) incorporation. Cells were incubated with each of the drugs at 7 different concentrations in triplicates for 6 days. The percentage of DNA replication was calculated as: % = 100% × (Absorbance treated)/(Absorbance untreated). (B, C) Cell viability in the olaparib‐resistant HCC1937 cell line was tested with CellTiter‐Glo® (Promega, USA) luminescent assay that quantified ATP in metabolically‐active cells after incubation with each of the drugs at 7–10 concentrations, in triplicates, for 6 days. The percent viability was calculated as: % = 100–(100 × (Luminescence untreated − Luminescence treated)/(Luminescence untreated – Luminescence blank)). (D) Cell viability of bovine vascular smooth muscle cells was assessed as described in B, C, following incubation with the drugs at 6 different concentrations, in quadruplicate, for 3 days.

The effect of Au (I) drugs on BRCA‐mutated cell proliferation was tested in the UWB1.289 ovarian carcinoma cell line, in comparison with rucaparib [22], olaparib [23], and talazoparib [24]—established NAD^+^‐mimetic PARP‐1 and PARP‐2 inhibitors approved in the United States and Europe for the treatment of cancers with germline BRCA mutations. The 5‐bromo‐2′‐deoxyuridine (BrdU) incorporation assay was performed such that BrdU was incorporated into DNA of replicating cells by substituting thymidine, and detected with BrdU‐specific antibodies (Fig. 3). We found that Au(I) drugs were 10‐fold more effective *in cellula* than *in vitro* (Tables 1 and 2). This effect could be attributed to the use of the recombinant PARP‐1 enzyme in the *in vitro* study. Recombinant proteins usually do not have the same level of post‐translational modification as their native counterparts, often leading to discrepancies between *in vitro* IC_50_ and *in cellula* EC_50_. Another possible explanation could be that the cellular accumulation of Au(I)‐based drugs might persist longer inside the cell yielding a higher effective concentration than parib drugs.

**Table 2:** Half maximal effective concentration (EC_50_) of ‘parib’ inhibitors and Au (I)‐based drugs in two BRCA cell lines differing in drug sensitivity and vascular smooth muscle cells. EC_50_ was calculated via plotting and fitting in Origin™, *R* ^2^ ≥ 0.9, values are given as mean ± s.e.m. For the UWB1.289 cell line the values of % DNA replication are provided in Fig. 3A. For the HCC1937 cell line and vascular smooth muscle cells the values of % viability are provided in Fig. 3B–D.

Compound	EC_50_/nm DNA replication assay	EC_50_/nm viability assay	
	‘Parib’‐sensitive cell line UWB1.289	‘Parib’‐resistant cell line HCC1937	Vascular smooth muscle cells
Aurothioglucose	3.8 ± 0.01	12 277 ± 4857	> 130 000
Sodium aurothiomalate	1.4 ± 0.02	84 ± 13	108 768 ± 4940
Auranofin	12.4 ± 1.2	89 ± 2	2032 ± 132
Talazoparib	≥ 50	6886 ± 1142	≥ 130
Olaparib	5497 ± 174	15 986 ± 3646	Not tested
Rucaparib	> 10 000	> 30 000	Not tested
Doxorubicin	Not tested	35.8 ± 0.5	Not tested
Paclitaxel	Not tested	< 10	105 ± 3

We observed substantial disparities in EC_50_ between ‘parib’ drugs (Fig. 3A and Table 2). It was previously attributed to the talazoparib ability to trap PARP‐1 in DNA‐PARP complexes, which then turn into very toxic double‐strand breaks. For instance, in the chicken lymphoma DT40 cell line, talazoparib was found to be 100‐fold more potent in PARP‐1 ‘trapping’ than rucaparib and olaparib [25], because rucaparib and olaparib were not effective ‘trappers’. In the UWB1.289 cell line the rucaparib EC_50_ was previously shown to be around 11 μm, which is similar to the value found in our work and much higher than the EC_50_ for talazoparib in the same cell line [26].

Unexpectedly, auranofin inhibited DNA replication almost as potently as talazoparib, aurothioglucose, and sodium aurothiomalate (Fig. 3A and Table 2), although its action could not be attributed solely to PARP‐1 inhibition, because it is not a potent PARP inhibitor (Fig. 2). Auranofin has several known protein targets and physiological effects in cells, including inhibition of thioredoxin reductase [27] and proteasome deubiquitinases [28], impediment of mitochondrial oxidative phosphorylation [29], and Ca^2+^ overload with subsequent apoptosis [30]. Despite auranofin having several known modes of action in the cancer cell, each of them individually has an IC_50_ in the micromolar range (including inhibition of PARP‐1 DNA‐dependent activity, Table 1), which is far too high when compared with EC_50_ = 12.4 nm found in our study (Fig. 3A and Table 2). Most likely, auranofin disrupted multiple pathways, leading to a cumulative effect and subsequent arrest of DNA replication at nanomolar concentrations.

Thus, we found that in BRCA‐mutated cell lines, aurothioglucose and sodium aurothiomalate were effective inhibitors of DNA replication (Fig. 3A) and that their half‐maximal effective concentration (EC_50_) was between 3 and 5 nm, which was one order of magnitude more effective than talazoparib and at least three orders of magnitude more effective, than olaparib and rucaparib (Table 2).

### *In vitro* safety of Au (I) drugs

The *in vitro* safety of aurothio‐compounds—specifically their effects on cell viability—was assessed using the ‘parib’‐resistant ductal carcinoma cell line HCC1937. This cell line carries mutations in three tumor suppressor genes: BRCA1, TP53, and PTEN; yet, it is known to be more resistant to olaparib compared to the HeLa cell line, which lacks a BRCA mutation [31]. Consistent with this, we found that HCC1937 cells were less sensitive to all ‘parib’ inhibitors than the UWB1.289 cell line, with EC_50_ values increasing by 100‐fold for talazoparib and approximately fivefold for both rucaparib and olaparib (Table 2).

Given that the cellular uptake mechanisms of talazoparib and olaparib/rucaparib involve different transporters [32, 33], we hypothesized that, in HCC1937 cells, the resistance mechanism circumvents PARP‐1–mediated DNA repair entirely. Under such conditions, aurothioglucose could only overcome PARP resistance and induce cell death if it also inhibited other essential zinc finger proteins involved in cellular survival. However, the EC_50_ of aurothioglucose shifted together with those of the ‘paribs’, suggesting its selectivity towards PARP‐1 in the cell (Fig. 3B and Table 2).

The EC_50_ of sodium aurothiomalate also increased in the HCC1937 cell line compared to the UWB1.289 cell line, but to a lesser extent than that of aurothioglucose or the ‘paribs’. This suggests that sodium aurothiomalate exhibits considerable cellular toxicity independent of PARP‐1 inhibition (Fig. 3C and Table 2). This effect may be attributed to the well‐documented broad cytotoxicity of aurothiomalate, a known side effect during its use in rheumatoid arthritis treatment. Nevertheless, sodium aurothiomalate was less toxic than doxorubicin and paclitaxel—two chemotherapeutic agents with known broad cellular toxicity that are still widely used in cancer therapy.

Auranofin was highly effective in killing HCC1937 cells, likely due to its ability to disrupt multiple cellular pathways (as discussed above), rather than through selective inhibition of PARP‐1.

Cancer drugs are often delivered to tumors via the bloodstream, which means the normal functioning of endothelial and vascular smooth muscle cells in blood vessels can be affected [34]. This may lead to conditions such as hypertension, thrombosis, and atherosclerosis. Therefore, it was important for us to demonstrate that the toxicity of aurothioglucose and sodium aurothiomalate did not exceed that of conventional drugs.

We found that aurothioglucose and sodium aurothiomalate were the least toxic to vascular smooth muscle cells compared with auranofin, talazoparib, and paclitaxel (Fig. 3D and Table 2), with the caveat that the cells used were of bovine rather than human origin. However, we have used bovine VSMCs in our research for years as a reliable model of human vascular diseases and showed that there were no substantial differences in their sensitivity to cancer drugs [10].

## Discussion

Aurothioglucose and sodium aurothiomalate are classified as disease‐modifying antirheumatic drugs, which suppress the progression of rheumatoid arthritis through multiple mechanisms. Rheumatoid arthritis is a chronic inflammatory disease characterized by the infiltration of phagocytes and leukocytes into synovial tissue, leading to joint swelling and the destruction of cartilage and bone. At the immune level, gold compounds are taken up by macrophages, where they inhibit antigen processing [35, 36]. At the effector level, Au(I) inhibits cysteine proteases such as cathepsins K and S, as well as matrix metalloproteinases including collagenase [37]. At the transcriptional level, Au(I) compounds downregulate a range of inflammatory genes by inhibiting the transcription factors NF‐κB and AP‐1 (Jun/Fos) [35, 38]. However, despite the well‐established role of PARP‐1 activation in the development of various inflammatory conditions—including pulmonary inflammation and cardiovascular disease—PARP‐1 inhibition by Au(I) compounds has never been considered among their mechanisms of action in rheumatoid arthritis.

In the mid‐1980s, auranofin was shown to inhibit cancer cell growth *in vitro*, and since then, multiple mechanisms of action have been proposed. These include inhibition of thioredoxin reductase [27], proteasome deubiquitinases [28], interference with mitochondrial phosphorylation [29], and induction of Ca^2+^ overload [30]. Yet, inhibition of PARP‐1 by Au(I) compounds has never been explored as a potential mechanism of cancer suppression.

Here we report that the well‐known Au(I) complexes used for decades in the treatment of rheumatoid arthritis are effective PARP‐1 inhibitors (Table 1 and Fig. 2). In the PARP‐1 zinc finger sites, the Zn^2+^ ion is coordinated in a tetrahedral geometry by a combination of cysteine with or without histidine residue(s), forming a Cys_2_HisCys coordination in the F1 and F2 fingers, and Cys_4_ in F3. Conditional dissociation constants for Zn(II) complexes in PARP‐1 were found at physiological pH to be 26 ± 4 nm for F1 and 4 ± 1 pM for F2. The former is indicative of a possibility that under cellular conditions F1 might exist in a ‘metal‐free’ state [39]. Au(I) and (III) have been reported to interact with cysteine residues of various zinc fingers leading to the formation of gold fingers. Examples include the reaction of aurothiomalate (Au(I)) with the third Cys_2_His_2_ zinc finger in the transcription factor Sp1. It was estimated that this classical zinc finger transcription factor has an apparent fourfold higher affinity for Au^+^ compared to Zn^2+^ [40]. Au (III) compounds were also reported to react with the second Cys_2_HisCys zinc finger of the HIV nucleocapsid protein, leading to the release of Zn^2+^ and the formation of Au_2_‐peptide and Au_4_‐peptide adducts [41]. The direct inhibition of PARP‐1 activity has been shown for Au(III) complexes along with the formation of a gold adduct in a peptide containing the F1 domain of PARP‐1 [42]. However, although IC_50_ values of the Au(III) complexes are as low as 7–8 nm, they cannot be used for *in vivo* studies due to their extreme cytotoxicity [42]. This is not the case for sodium aurothiomalate and aurothioglucose which have been used for decades in rheumatoid arthritis patients. Here, we have shown that these two compounds inhibit the DNA‐dependent activity of PARP‐1 in the tens to hundreds nanomolar range of concentration. At the same time, the DNA‐independent activity of PARP‐1 is preserved (Table 1 and Fig. 2), suggesting that both drugs act as zinc ejectors [43], disrupting the PARP‐1 zinc‐finger structures, and consequently, the PARP‐1‐ssb DNA binding.

There are general safety concerns around the selectivity of drugs that target zinc fingers because zinc fingers are ubiquitous in the cell and play important roles in many cellular processes such as transcription regulation, signal transduction, DNA repair, cell cycle regulation, cell migration, etc. Indeed, there are ca. 3000 zinc‐binding proteins in the human genome, and the majority are zinc finger proteins [44], so if a zinc ejector unintentionally binds to the wrong protein(s), it may have a resonating toxic effect all over the body. However, our data suggest that at least in the ‘parib’ resistant BRCA cell line HCC1937 (Table 2 and Fig. 3B), aurothioglucose exhibits a higher degree of selectivity for PARP‐1. In the same cell line aurothiomalate and auranofin demonstrate some general toxicity (Table 2 and Fig. 3C), though both are less toxic than conventional chemotherapeutic drugs doxorubicin and paclitaxel. Aurothioglucose and sodium aurothiomalate were also found to be the least toxic to vascular smooth muscle cells compared with the conventionally used drugs talazoparib and paclitaxel (Table 2 and Fig. 3D), suggesting—together with similar results in the ‘parib’‐resistant BRCA cell line HCC1937—a potentially higher level of systemic tolerance to these compounds.

It is now widely accepted that complete inhibition of a vital pathway in cancer cells can eliminate the most sensitive clones within a tumor population, but simultaneously promote the proliferation of clones that either possess a pre‐existing resistant phenotype or acquire resistance during treatment. Therefore, using a selective zinc‐ejector drug—rather than a NAD^+^‐mimetic inhibitor—could offer a strategic advantage. Such a compound would partially inhibit PARP‐1 by disrupting its DNA damage response (DDR) activity while preserving its basal (DNA‐independent) functions. In this context, the zinc‐finger inhibition may reduce the selective pressure on the tumor population to develop resistance, as the biological drive to escape PARP‐1 inhibition would be comparatively weak.

Furthermore, when drug resistance arises from increased drug efflux—such as the upregulation of ABC transporters during rucaparib or olaparib treatment [45] —switching to an alternative class of inhibitors may become essential. In this context, selective zinc finger inhibitors of PARP‐1 present a promising option. The discovery of selective and potent PARP‐1 inhibitors among existing drugs with well‐established safety profiles, known dosing, and characterized side effects presents new opportunities for anticancer drug testing, offering faster development timelines and reduced costs.

## Author contributions

UB conceived the idea, conducted experiments, wrote the main manuscript text, and prepared figures. MJD oversaw the project and manuscript preparation.

## Data Availability

The data that support the findings of this study are available upon request from both authors.
